# Self-esteem as a determinant of sexual function in systemic lupus erythematosus patients: a cross-sectional study

**DOI:** 10.1186/s42358-026-00570-y

**Published:** 2026-07-25

**Authors:** Daniela Marengo-Rodríguez, María Espinosa-León, Monserrat Ibarra-Velasco-Siles, Paola Vázquez-Cárdenas, Erik Cimé-Aké, Jonathan Campos-Guzmán, Ana Barrera-Vargas, Javier Merayo-Chalico

**Affiliations:** 1 Department of Immunology and Rheumatology, Instituto Nacional de Ciencias Médicas y Nutrición Salvador Zubirán, Avenida Vasco de Quiroga 15, Belisario Domínguez Sección XVI, Tlalpan, Mexico City, C.P. 14080 Mexico; 2 Department of Molecular Biology and Histocompatibility, Hospital General Dr. Manuel Gea González, Mexico City, Mexico; 3 Department of Medical Education, Instituto Nacional de Ciencias Médicas y Nutrición Salvador Zubirán, Mexico City, Mexico

**Keywords:** Sexual function, Self-esteem, Systemic lupus erythematosus, Patient-reported outcomes

## Abstract

**Background:**

The correlation between self-esteem and sexual function in systemic lupus erythematosus (SLE) patients remains unexplored. The objective was to assess the association between body self-esteem, global self-esteem, and sexual function in patients with SLE.

**Methods:**

A cross-sectional study was conducted at a tertiary care center. We included patients aged 18 and older who met the EULAR/ACR criteria for SLE. Body self-esteem was measured using the Body Self-Esteem Scale (BSES), while global self-esteem was assessed with the Rosenberg Self-Esteem Scale (RSE). Sexual function was evaluated using the Changes in Sexual Functioning Questionnaire Short-Form questionnaire (CSFQ-14). Sexual dysfunction (SD) was defined as a total CSFQ-14 score of less than 41 points for women and less than 47 points for men. We assessed disease activity using the SLEDAI-2 K, and damage accrual with the SLICC damage index.

**Results:**

A total of 280 patients were included, the majority of whom were female (88.6%). The median age was 39 years (IQR 31 to 49). The median SLEDAI-2 K score was 2 (IQR 0 to 4), and the SLICC damage index was 0 (IQR 0 to 1). A total of 147 patients (52.5%) were classified as having SD. The BSES score showed a significant correlation with the total CSFQ-14 score (rho = 0.364, *p* < 0.001). Similarly, the RSE subcomponents, self-competence (rho = 0.262, *p* < 0.001) and self-liking (rho = 0.274, *p* < 0.001) showed both a significant positive correlation with the total CSFQ-14 score. Patients with SD compared to those without SD had significantly lower BSES scores, lower self-competence scores and lower self-liking scores. Female sex (OR 2.6, 95% CI 1.2 to 6.0, *p* = 0.026), lower leukocyte count (OR 1.2, 95% CI 1.0 to 1.3, *p* = 0.046), and lower BSES score (OR 1.9, 95% CI 1.8 to 2.1, *p* < 0.001) were independent risk factors for sexual dysfunction.

**Conclusions:**

This is the first study to evaluate self-esteem as a determinant of sexual function in SLE patients. Given the significant impact of self-esteem on sexual dysfunction, its relationship should be assessed during rheumatology evaluations.

**Clinical trial number:**

Not applicable.

## Background

Systemic lupus erythematosus (SLE) is a chronic autoimmune disease that affects multiple organs [1], and the accumulation of damage is strongly associated with increased patient mortality [2]. SLE patients not only experience significant physical deterioration but also report worse mental well-being than patients with other autoimmune diseases, such as rheumatoid arthritis [3]. SLE significantly impacts multiple aspects of a patient’s life, leading to psychological impairments such as low self-esteem and depression, as well as affecting sexual function and health-related quality of life [4, 5]. Sexual function encompasses issues that affect any phase of the sexual response cycle, ultimately impeding sexual satisfaction [6]. In males, this includes concerns like erectile dysfunction, abnormal ejaculation, and low libido [7], while females may encounter disorders related to desire, orgasm, arousal, and sexual pain [8]. Notably, the frequency of impaired sexual function among SLE patients surpasses that in healthy individuals [9, 10]. Patients with SLE often exhibit negative attitudes towards their body image [11]. Self-esteem is the perception and valuation that an individual holds for oneself, influenced by various determinants, both direct and indirect [5]. Previous studies in patients with rheumatoid arthritis and SLE have found that health status is directly related to self-esteem, but not to body image [12]. Although sexual dysfunction and its associated factors have been extensively studied in SLE patients [13, 14], the direct correlation between self-esteem and sexual function in SLE patients remains unexplored. Therefore, our study aims to assess the association between body self-esteem, global self-esteem, and sexual function in SLE patients, and to explore how these factors relate to clinical, demographic, and disease-specific variables.

## Methods

### Design and patients

We conducted a cross-sectional study at a tertiary care center in Mexico City between February 2022 and February 2023. The study included sexually active Mexican mestizo men and women aged 18 years and older, who had a partner within the past four weeks and met the 2019 EULAR/ACR classification criteria for SLE [15]. Patients with other rheumatologic diseases (e.g., antiphospholipid syndrome, Sjögren’s disease, rheumatoid arthritis) were excluded, as well as those with conditions potentially affecting sexual function, including cancer, chronic infections, untreated hypothyroidism, hypogonadism, liver disease, pregnancy, hormonal therapy, or a history of urological or pelvic procedures, in order to minimize confounding factors. The sample size was determined based on the previously reported prevalence of sexual dysfunction in SLE [16]. We performed a simple random sampling.

### Outcomes

Self-esteem was assessed using two distinct scales: The Body Self-Esteem Scale (BSES) [17] and Rosenberg’s Self-Esteem Scale (RSE) [18]. The BSES is a tool developed and validated in Spanish that consists of 26 items with a unidimensional structure that evaluates body self-esteem. Of these, 20 items primarily assess the cognitive aspect, or Body Satisfaction, while six items focus on the emotional aspect, or Body Attractiveness. Responses are rated on a Likert-type scale from 1 (very dissatisfied) to 10 (very satisfied). The total BSES score is obtained by adding the scores of all 26 items [17].

Global self-esteem was assessed using the Spanish-validated version of Rosenberg’s Self-Esteem Scale (RSE) [19], a 10-item scale measuring both positive and negative feelings about oneself. Responses are provided on a 4-point Likert scale. Agreement with the statements is rated as follows: low self-esteem responses include “disagree” or “strongly disagree” for items 1, 3, 4, 7, and 10, and “strongly agree” or “agree” for items 2, 5, 6, 8, and 9. The total score, ranging from 10 to 40, is calculated by adding the individual item scores after reverse-scoring the negatively worded items. Additionally, the RSE was analyzed according to its two subcomponents: self-competence (items 3, 4, 5, 7, and 9) and self-liking (items 1, 2, 6, 8, and 10), as previously described [20]. In both the BSES and RSE, a higher score indicates better self-esteem.

Sexual function was assessed using the Spanish version of the Changes in Sexual Functioning Questionnaire Short-Form (CSFQ-14) [21], which includes 14 questions evaluating total and subscale scores related to sexual desire/frequency, sexual desire/interest, sexual pleasure, sexual arousal/excitement, and sexual orgasm/completion, with scoring differences considered between men and women. The total score ranges from 14 to 70, with sexual dysfunction defined as a total score of less than 41 points for women and less than 47 points for men, as established by the questionnaire [22].

These questionnaires were completed privately by the participants without input from the recruiter. We collected demographic information, anthropometric data, disease activity history, comorbidities, laboratory measurements, antibody profiles, current medication use, and SLEDAI-2 K [23] and SLICC damage index (SDI) [24] scores. Active disease was defined as a SLEDAI-2 K score ≥ 6 points.

### Statistical analysis

Data was analysed using SPSS version 29.0 for Mac OS (IBM, Armonk, NY, USA). Descriptive statistics were used for variables, presented as percentage or medians with interquartile range. A comparison was conducted between groups of patients with and without sexual dysfunction (SD), as well as between males and females using the Chi-square, Fisher’s exact test and Wilcoxon rank-sum test. Spearman’s correlation was applied to analyse the correlation between self-esteem scores and SD. Forward stepwise binary logistic regression was performed to identify the independent factors for SD. In this regard, variables associated with SD in univariate analyses (*p* < 0.05) and those considered clinically relevant (age, major depressive disorder, SLEDAI-2 K, SDI, BSES and RSE scores) regardless of statistical significance were included in the multivariable logistic regression model. Statistical significance was set at a p-value < 0.05 with a 95% confidence interval (CI) using a two-tailed test.

## Results

A total of 280 SLE patients were included, with a median age of 39 years (IQR 31 to 49). The majority were female (88.6%) and had low SLEDAI-2 K (2 [IQR 0 to 4]) and SDI (0 [IQR 0 to 1]) scores. Based on the total CSFQ-14 score, 147 patients (52.5%) were classified as having SD. The SD group had a higher percentage of women compared to the group without SD (93.2% vs. 83.5%, *p* = 0.011). Both groups were comparable in terms of age, disease activity, and damage accrual. A total of 24 patients (8.6%) had an established diagnosis of major depressive disorder (MDD); no difference was found when comparing the number of patients with MDD between those with and without SD (9.5% vs. 7.5%, *p* = 0.550). Despite having leukocyte counts within the normal range, patients with SD exhibited significantly lower leukocyte counts (5.1 K/µL [IQR 3.6 to 5.9 K/µL] vs. 5.5 K/µL [IQR 4.2 to 6.8 K/µL], *p* = 0.004) and neutrophil counts (3.1 K/µL [IQR 2.3 to 3.7 K/µL] vs. 3.3 K/µL [IQR 2.6 to 4.3 K/µL], *p* = 0.014) compared to those without SD. However, no significant differences were observed between the two groups in hemoglobin, lymphocytes, platelets, creatinine, complement levels, anti-dsDNA antibodies, or current immunosuppressive treatment. Table 1 presents the main clinical and demographic characteristics of the SLE groups.

**Table 1:** Clinical and laboratory characteristics of included patients (*n* = 280)

Variables	Total *n* = 280	With SD *n* = 147	Without SD *n* = 133	*p*
*Clinical features*				
Age, years	39.0 (31.0–49.0)	39.0 (31.0–50.0)	38.0 (31.0–48.0)	0.270
**Female sex**,** n (%)**	**248 (88.6)**	**137 (93.2)**	**111 (83.5)**	0.011*
BMI	25.1 (22.2–28.3)	24.7 (22.0–27.0)	25.4 (22.5–29.0)	0.057
SLEDAI-2 K	2 (0–4)	2 (0–4)	2 (0–4)	0.391
SDI	0 (0–1)	1 (0–1)	0 (0–1)	0.266
*Laboratory findings*				
Hemoglobin, g/dL	13.7 (12.6–14.6)	13.6 (12.4–14.5)	13.8 (12.7–14.9)	0.071
**Leukocytes**,** K/µL**	**5.2 (3.9–6.3)**	**5.1 (3.6–5.9)**	**5.5 (4.2–6.8)**	0.004*
**Neutrophils**,** K/µL**	**3.1 (2.5-4.0)**	**3.1 (2.3–3.7)**	**3.3 (2.6–4.3)**	0.014*
Lymphocytes, K/µL	1.3 (0.9–1.8)	1.3 (0.8–1.7)	1.3 (1.0-1.8)	0.152
Platelets, K/µL	220.0 (180.0-268.0)	216.0 (182.0-264.0)	225.0 (175.3–268.0)	0.460
Creatinine, mg/dL	0.7 (0.6–0.9)	0.7 (0.6–0.8)	0.7 (0.6–0.9)	0.515
C3, mg/dL	113.0 (92.0-134.0)	108.0 (91.0-131.5)	117.0 (96.0-136.0)	0.087
C4, mg/dL	21.0 (13.0–28.0)	21.0 (13.0–27.0)	21.0 (12.0-28.3)	0.948
Anti-dsDNA, IU/mL	10.5 (5.9–52.1)	13.2 (5.6–71.0)	8.7 (6.3–38.6)	0.281
*Current immunosuppressive treatment*				
Prednisone dose, mg/day	0 (0–5)	0 (0–5)	0 (0–5)	0.409
Hydroxychloroquine, n (%)	195 (69.6)	104 (70.7)	91 (68.4)	0.672
Cyclophosphamide, n (%)	4 (1.4)	2 (1.4)	2 (1.5)	1.000
Methotrexate, n (%)	24 (8.6)	14 (9.5)	10 (7.5)	0.550
Azathioprine, n (%)	61 (21.8)	34 (23.1)	27 (20.3)	0.567
Mycophenolate mofetil, n (%)	120 (42.9)	57 (38.8)	63 (47.4)	0.184
*Self-esteem*				
**Body Satisfaction score**	**164.0 (136.0-181.0)**	**160.0 (123.0-174.5)**	**168.0 (141.0-187.0)**	< 0.001*
**Body Attractiveness score**	**41.0 (30.0–50.0)**	**35.0 (25.0–48.0)**	**48.0 (42.0–54.0)**	< 0.001*
**BSES score**	**206.0 (175.0-232.0)**	**196.0 (155.0-222.0)**	**218.0 (190.0-241.0)**	< 0.001*
**Self-competence score**	**18.0 (16.0–20.0)**	**17.0 (15.0–19.0)**	**19.0 (17.0–20.0)**	< 0.001*
**Self-liking score**	**15.0 (13.0–17.0)**	**14.0 (12.0–17.0)**	**16.0 (14.0–18.0)**	< 0.001*
RSE score	27.0 (25.0–29.0)	27.0 (25.0–29.0)	27.0 (25.0–29.0)	0.975

Data are expressed as frequencies (percentage) or medians (interquartile ranges). The p values correspond to comparisons between patients with and without sexual dysfunction. Sexual dysfunction was defined as a total CSFQ-14 score of less than 41 points for women and less than 47 points for men. Body Satisfaction and Body Attractiveness correspond to subdomains of the Body Self-Esteem Scale (BSES). Self-competence and Self-liking correspond to subcomponents of the Rosenberg’s Self-Esteem Scale (RSE). Statistical analysis: Chi-square and Wilcoxon rank-sum test

SD: sexual dysfunction, BMI: body mass index, SLEDAI-2 K: Systemic Lupus Erythematosus Disease Activity Index 2000, SDI: SLICC/ACR Damage Index

*Statistically significance

**Fig. 1:**
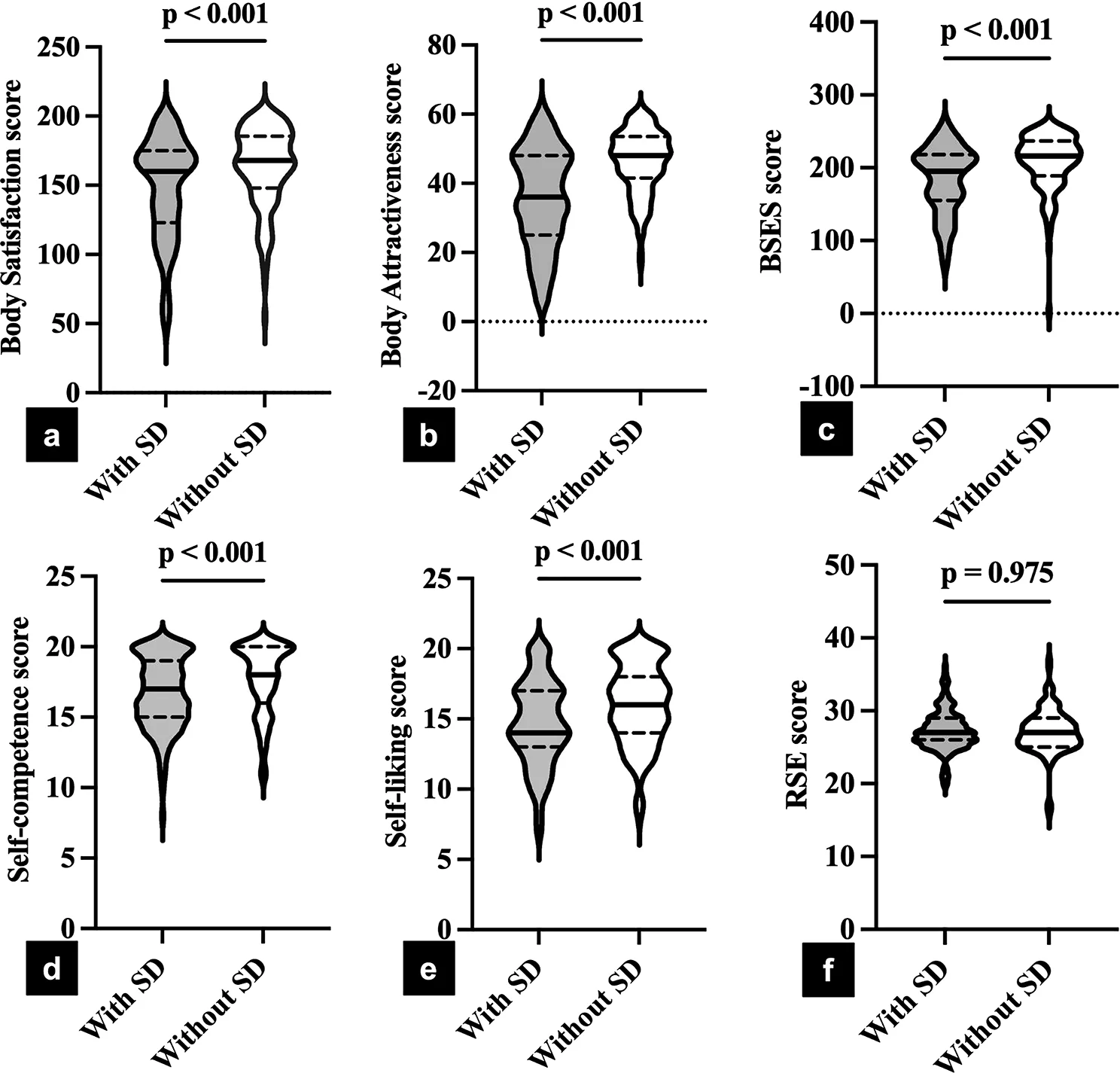
Self-esteem scales according to sexual function in SLE patients. (**a**) Body Satisfaction score; (**b**) Body Attractiveness score; (**c**) BSES score; (**d**) Self-competence score; (**e**) Self-liking score; (**f**) RSE score. Sexual dysfunction was defined as a total CSFQ-14 score of less than 41 points for women and less than 47 points for men. Body Satisfaction and Body Attractiveness correspond to subdomains of the Body Self-Esteem Scale (BSES). Self-competence and Self-liking correspond to subcomponents of the Rosenberg’s Self-Esteem Scale (RSE). Statistical analysis: Wilcoxon rank-sum test. SD: sexual dysfunction, BSES: Body Self-Esteem Scale, RSE: Rosenberg’s Self-Esteem Scale

**Fig. 2:**
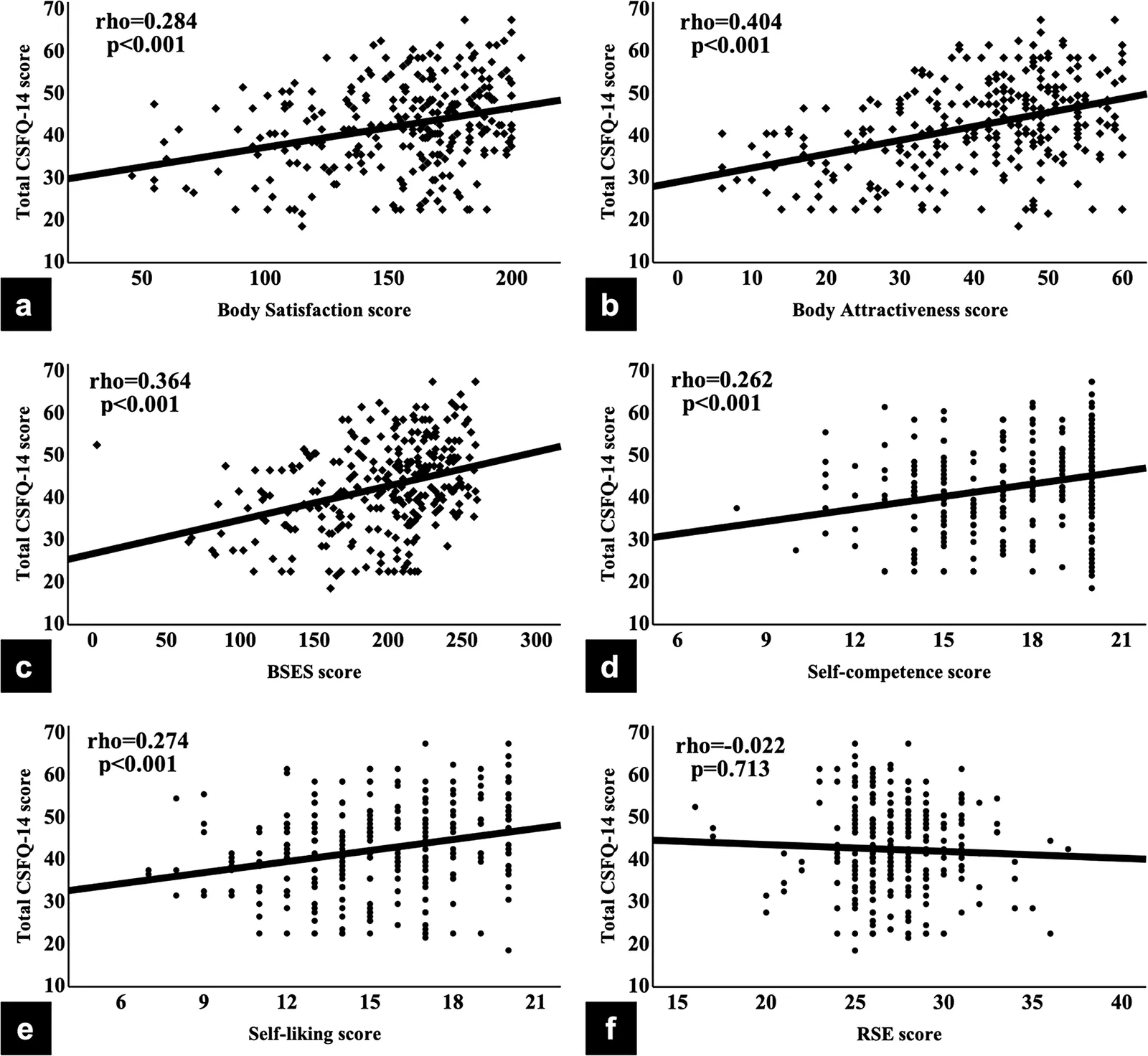
Correlations between self-esteem scores and sexual function in SLE patients. (**a**) Body Satisfaction score and total CSFQ-14 score; (**b**) Body Attractiveness score and total CSFQ-14 score; (**c**) BSES score and total CSFQ-14 score; (**d**) Self-competence score and total CSFQ-14 score; (**e**) Self-liking score and total CSFQ-14 score; (**f**) RSE score and total CSFQ-14 score. Body Satisfaction and Body Attractiveness correspond to subdomains of the Body Self-Esteem Scale (BSES). Self-competence and Self-liking correspond to subcomponents of the Rosenberg’s Self-Esteem Scale (RSE). Statistical analysis: Spearman’s correlation. BSES: Body Self-Esteem Scale, RSE: Rosenberg’s Self-Esteem Scale, CSFQ-14: Changes in Sexual Functioning Questionnaire-14

When comparing self-esteem scale scores based on the presence of SD, patients with SD had significantly lower Body Satisfaction (160.0 [IQR 123.0 to 175.0] vs. 168.0 [IQR 148.0 to 185.5], *p* < 0.001), lower Body Attractiveness (36.0 [IQR 25.0 to 48.0] vs. 48.0 [IQR 41.5 to 53.5], *p* < 0.001), and lower BSES scores (195.0 [IQR 155.0 to 218.0] vs. 216.0 [IQR 189.0 to 237.0], *p* < 0.001) compared to those without SD. Although no differences were found in the total RSE score between patients with and without SD (27.0 [IQR 26.0 to 29.0] vs. 27.0 [IQR 25.0 to 29.0], *p* = 0.975), both self-competence and self-liking scores were significantly lower in patients with SD compared to those without (17.0 [IQR 15.0 to 19.0] vs. 18.0 [IQR 16.0 to 20.0], *p* < 0.001, and 14.0 [IQR 13.0 to 17.0] vs. 16.0 [14.0 to 18.0], *p* < 0.001], respectively), as shown in Fig. 1. Among all patients, there was a significant positive correlation between the Body Satisfaction score and the total CSFQ-14 score (rho = 0.284, *p* < 0.001), as well as between the Body Attractiveness score and the total CSFQ-14 score (rho = 0.404, *p* < 0.001). Similarly, the total BSES score showed a significant correlation with the CSFQ-14 score (rho = 0.364, *p* < 0.001). No significant correlation was observed between the total RSE score and the total CSFQ-14 score (rho=-0.022, *p* = 0.713). However, when the RSE was analyzed according to its subcomponents, both self-competence (rho = 0.262, *p* < 0.001) and self-liking (rho = 0.274, *p* < 0.001) showed a significant positive correlation with the total CSFQ-14 score, as shown in Fig. 2.

**Fig. 3:**
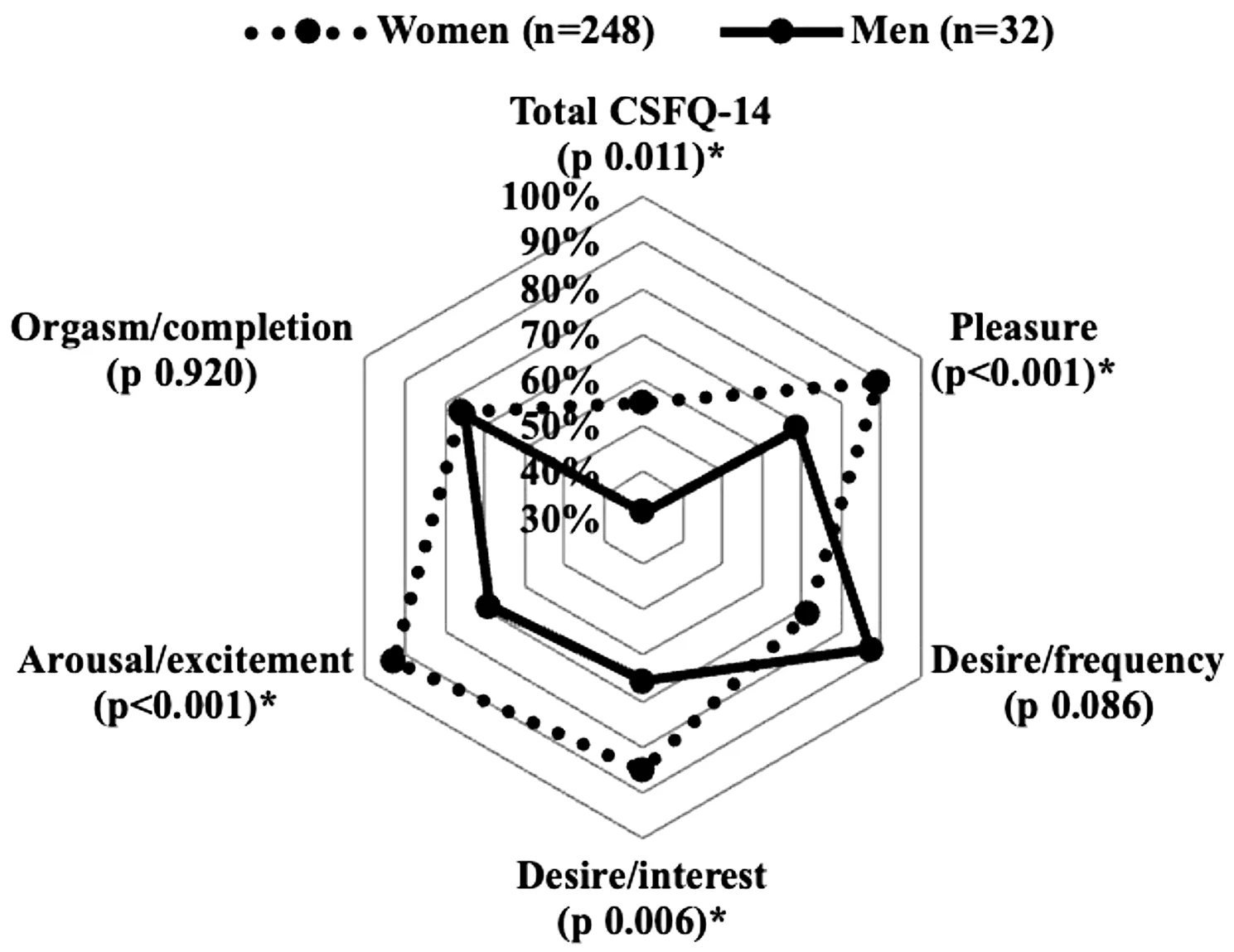
Sexual function by domains according to sex in SLE patients. Data represents the percentage of sexual dysfunction according to every Changes in Sexual Functioning Questionnaire Short-Form-14 (CSFQ-14) sub-domain and the total score. Pleasure, desire/frequency, desire/interest, arousal/excitement and orgasm/completion are sub-domains of the CSFQ-14. Statistical analysis: Chi-square or Fisher’s exact test. *Statistically significance

Self-esteem scores were compared between sexes, showing no significant differences. Notably, women exhibited a higher prevalence of SD compared with men (55.2% vs. 32.3%, *p* = 0.011). When analyzing the RSE according to its two subcomponents, no significant differences were found between men and women in either self-competence or self-liking (Table 2). A sub-analysis of the CSFQ-14 domains revealed that women had a significantly higher prevalence of SD in the pleasure (89.5% vs. 68.8%, *p* < 0.001), desire/interest (85.1% vs. 65.6%, *p* = 0.006), and arousal/excitement (92.7% vs. 68.8%, *p* < 0.001) domains compared to men, as illustrated in Fig. 3.

**Table 2:** Self-esteem and sexual dysfunction in SLE patients (*n* = 280)

Variables	Total, *n* = 280	Women, *n* = 248	Men, *n* = 32	*p*
Body Satisfaction score	164.0 (136.0-181.0)	164.0 (134.0-178.8)	169.0 (144.3–187.0)	0.128
Body Attractiveness score	43.5 (32.3–50.0)	43.0 (32.0–50.0)	46.0 (36.3–49.8)	0.542
BSES score	206.5 (169.0-227.5)	206.0 (167.0-225.0)	218.0 (182.5–234.0)	0.145
Self-competence score	18.0 (15.0–20.0)	18.0 (15.0–20.0)	18.0 (15.0–20.0)	0.528
Self-liking score	15.0 (13.0–17.0)	15.0 (13.0–17.0)	16.0 (14.3–19.0)	0.072
RSE score	27.0 (26.0–29.0)	27.0 (26.0–29.0)	26.0 (25.0–28.0)	0.163
**Sexual Dysfunction**,** n (%)†**	**147 (52.5)**	**137 (55.2)**	**10 (32.3)**	0.011*
**Total CSFQ-14 score**	**41.0 (35.0–49.0)**	**41.0 (34.3–48.0)**	**56.0 (46.3–60.8)**	< 0.001*

Data are expressed as proportions (percentages) or medians (interquartile ranges). The p values correspond to comparisons between women and men. Body Satisfaction and Body Attractiveness correspond to subdomains of the Body Self-Esteem Scale (BSES). Self-competence and Self-liking correspond to subcomponents of the Rosenberg’s Self-Esteem Scale (RSE). Statistical analysis: Chi-square or Fisher’s exact test were used for nominal variables, the Wilcoxon rank-sum test was used for numerical variables

BSES: Body Self-Esteem Scale, RSE: Rosenberg’s Self-Esteem Scale, CSFQ-14: Changes in Sexual Functioning Questionnaire-14

†Based on the total CSFQ-14 score

*Statistically significance

**Fig. 4:**
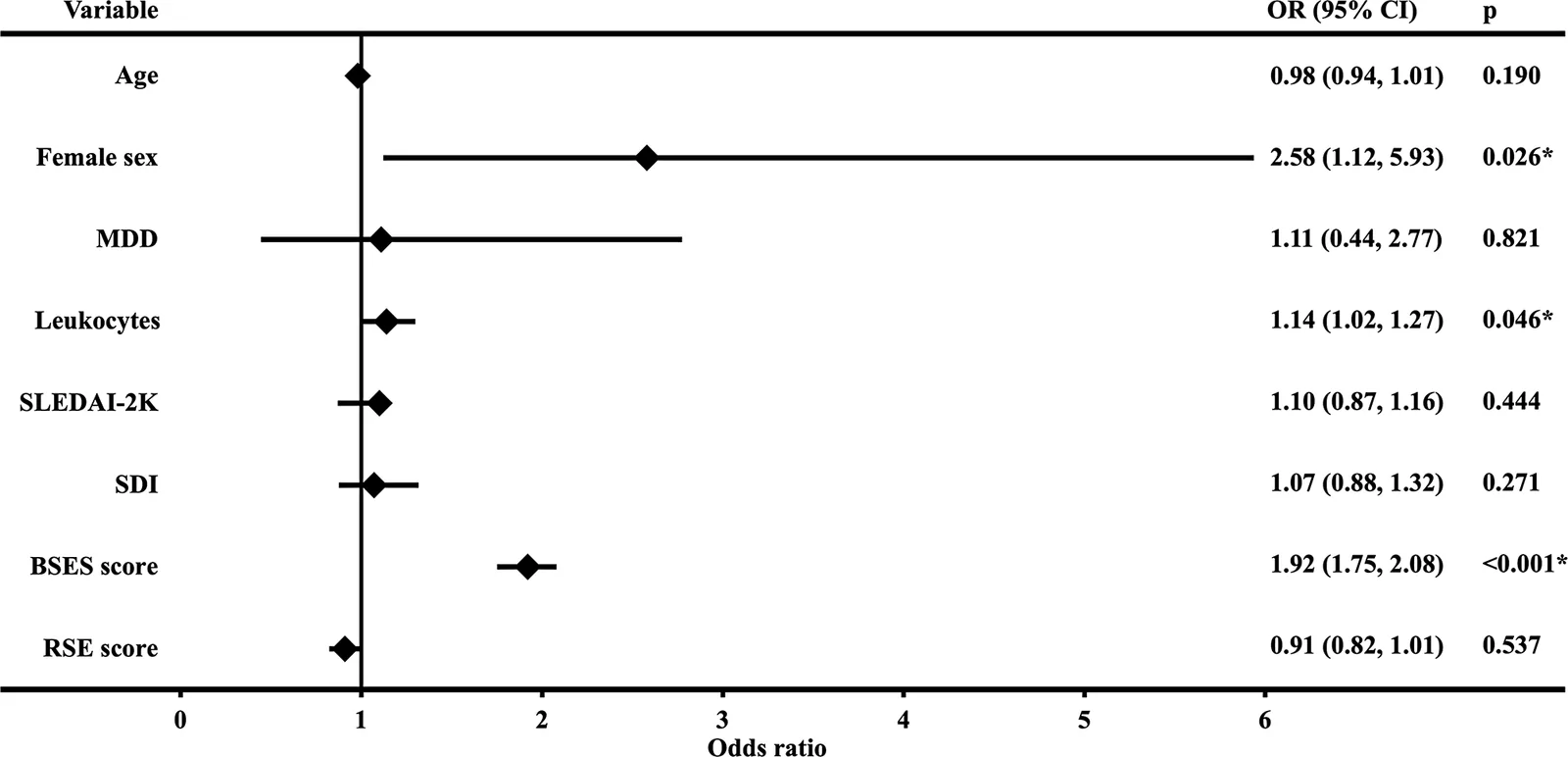
Factors associated with sexual dysfunction in SLE patients. Data represent the OR and 95% CI for each variable included in the multivariate analysis. Variables associated with sexual dysfunction in univariate analyses (female sex, leukocytes, BSES score) and those considered clinically relevant (age, major depressive disorder, SLEDAI-2 K, SDI, and RSE scores) regardless of statistical significance were included in the multivariable logistic regression model. Statistical analysis: Forward stepwise binary logistic regression. MDD: major depressive disorder, SLEDAI-2 K: Systemic Lupus Erythematosus Disease Activity Index 2000, SDI: SLICC/ACR Damage Index, BSES: Body Self-Esteem Scale, RSE: Rosenberg’s Self-Esteem Scale. *Statistically significance

Multivariate analysis showed that female sex (OR 2.6, 95% CI 1.2 to 6.0, *p* = 0.026), lower leukocyte count (OR 1.2, 95% CI 1.0 to 1.3, *p* = 0.046), and lower BSES score (OR 1.9, 95% CI 1.8 to 2.1, *p* < 0.001) were independent risk factors for SD, as depicted in Fig. 4.

## Discussion

This study evaluates self-esteem as a determinant of sexual function in patients with SLE. Our findings indicate a notably high prevalence of SD in this population, consistent with previous studies reporting elevated rates of sexual impairments in autoimmune diseases [25]. Importantly, we identified female sex, leukocyte count, and low body self-esteem, as assessed by the Body Self-Esteem Scale, as independent predictors of SD.

Our results reveal an SD prevalence of more than a half of our patients, comparable to the high prevalence reported in patients with type 2 diabetes [26]. However, while in type 2 diabetes such rates are typically observed in patients aged 60 or older with associated complications [27], our cohort included younger patients with low disease activity and minimal damage accrual. This discrepancy underscores the need to explore how psychological factors, such as body perception, significantly impact sexual function even in the absence of severe clinical manifestations.

Self-esteem, a multidimensional construct influencing mental health [28], encompasses domains such as body, social, achievement, and identification self-esteem [29]. In this study, we found that low body self-esteem, rather than global self-esteem, was significantly associated with SD. These findings align with previous studies indicating that altered body image negatively impacts sexual health in SLE patients [16]. Factors such as fatigue, depression, comorbidities, and disease activity have been identified as predictors of altered body image [30]. Additionally, cutaneous manifestations such as alopecia and visible lesions correlate with poorer body image and quality of life, highlighting the need to address these areas in clinical care [31, 32]. It appears that not only the clinical manifestations of the disease influence the self-esteem of patients living with lupus, as many patients reported feeling trapped in a state of constant uncertainty and limitations in their physical and social functioning [33].

An additional relevant finding was that body self-esteem, as measured by the BSES, was independently associated with sexual dysfunction, whereas global self-esteem assessed by the RSE was not. This difference may be explained by the conceptual and psychometric distinctions between both instruments. The BSES specifically evaluates body-related perceptions, which are more directly linked to sexual function, while the RSE measures a broader construct of global self-esteem. Previous studies have demonstrated a strong relationship between body image and sexual function [34, 35]. An interesting finding regarding the lack of association between the total RSE score and sexual function, was that there were significant associations observed when analyzing its subcomponents. Both self-competence and self-liking were positively correlated with CSFQ-14 scores and were lower in patients with SD. This suggests that the multidimensional structure of global self-esteem may not be fully captured by the total RSE score, potentially masking domain-specific associations. Previous literature has highlighted that self-competence and self-liking represent distinct dimensions of self-esteem, which may differentially relate to clinical outcomes [36, 37]. Our findings support the importance of analyzing these subcomponents separately, as they appear to be more sensitive in detecting associations with sexual function.

Interestingly, medications, including glucocorticoids—which frequently affect physical appearance in other rheumatic diseases—showed no association with SD or self-esteem in this study [38]. This suggests that patients’ perceptions of their illness and limitations may play a more significant role in sexual function than sociodemographic or clinical factors [39]. This finding reinforces the importance of a biopsychosocial approach to managing SLE patients, considering that SD may be mediated by psychological and social elements rather than strictly clinical manifestations.

Another relevant finding was that female sex represented an independent risk factor for SD, particularly in domains such as pleasure, desire/interest, and arousal. This result is consistent with the predominance of young women in SLE populations [40] and the greater disease severity reported in Latin American populations [41]. Recent studies have documented the negative impact of SLE on sexual quality of life, including aspects such as desire, arousal, and pain [42]. Moreover, low body self-esteem could contribute to prolonged preconception periods in women with SLE, as observed in other autoimmune diseases [43]. Regarding men, although they represented a minority in our cohort, significant rates of SD were also observed. This was similar with previous research associating erectile dysfunction with recent glucocorticoid use and lymphopenia in men with SLE [44, 45]. Although previous studies have reported gender differences between self-competence and self-liking, such differences were not observed in our patients [46]. Differences in SD prevalence and self-esteem between sexes may be attributed to neurobiological changes [47] and sociocultural influences [48], warranting further investigation.

Our study also suggests that interventions aimed at improving body self-esteem could benefit quality of life and sexual function in SLE patients. Although evidence on managing SD in SLE is limited, a recent systematic review recommended strategies to improve body image and self-concept, including early screening for depression, anxiety, and relationship problems, as well as supportive products such as cosmetics [49]. Moreover, in other populations, such as breast cancer patients, physical and psychoeducational interventions have shown improvements in self-esteem and aspects of sexual function, such as pleasure [50, 51]. Pharmacological interventions, such as sildenafil, have also demonstrated benefits in enhancing self-esteem and interpersonal relationships in men with erectile dysfunction [52]. Thus, it is crucial to develop comprehensive strategies to address these aspects in SLE patients.

Finally, while lower leukocyte counts were identified as a risk factor for SD, these values remained within the normal range and their clinical significance is limited. However, this may represent an area for future exploration to understand how hematological parameters could impact sexual health.

This study has several limitations. Its cross-sectional design precludes establishing causal relationships between psychological variables and their impact on sexual function. Data relied solely on patient self-reports, without assessments from specialized professionals such as sexologists or psychologists. An additional limitation of this study is that only sexually active patients who had a partner within the previous four weeks were included. This criterion may have introduced a selection bias, as patients without recent sexual activity were not evaluated. Importantly, the absence of sexual activity may itself represent a severe manifestation of sexual dysfunction. Therefore, our findings may underestimate the true prevalence and impact of SD in patients with SLE. Other factors not fully captured in our study may influence SD. Although we excluded conditions known to affect sexual function and accounted for depression, other relevant comorbidities such as anxiety, fibromyalgia, hormonal factors, and metabolic disorders were not systematically evaluated. In addition, detailed information on SLE clinical manifestations, antiphospholipid antibodies, and socioeconomic or psychosocial variables (such as relationship status, number of partners, or sexual preferences) was not available. These factors may act as potential confounders and could influence sexual function. Therefore, our findings should be interpreted with caution, and future studies incorporating a more comprehensive assessment of these variables are warranted. The study was conducted at a single center with a predominantly Latin population, which may limit the generalizability of the findings. Furthermore, the low representation of men, although reflective of SLE epidemiology, may constrain the applicability of the results. Despite these limitations, this is the first study to evaluate the association between self-esteem and SD in SLE patients using validated tools, which constitutes a key strength factor.

## Conclusions

In conclusion, low body self-esteem is an independent risk factor for sexual dysfunction in SLE patients, particularly in women. Given its significant impact, self-esteem should be evaluated during rheumatology consultations as part of a holistic approach to patient care. This reflects not only the influence of SLE as a chronic disease but also represents a key opportunity to improve patients’ overall well-being. Integrating sexual health through screening and multidisciplinary interventions should be prioritized to enhance patient-centered care and promote self-care. 

## Data Availability

No datasets were generated or analysed during the current study.

## References

[1] Siegel CH, Sammaritano LR. Systemic lupus erythematosus: a review. JAMA. 2024;331(17):1480–91. 10.1001/jama.2024.2315 38587826

[2] Segura BT, Bernstein BS, McDonnell T, et al. Damage accrual and mortality over long-term follow-up in 300 patients with systemic lupus erythematosus in a multi-ethnic British cohort. Rheumatology (Oxford). 2020;59(3):524–33. 10.1093/rheumatology/kez292 PMC8414923 31377781

[3] Wolfe F, Michaud K, Li T, et al. EQ-5D and SF-36 quality of life measures in systemic lupus erythematosus: comparisons with rheumatoid arthritis, non-inflammatory rheumatic disorders, and fibromyalgia. J Rheumatol. 2010;37(2):296–304. 10.3899/jrheum.090778 20032098

[4] Jin Z, Yang C, Xiao C, et al. Systemic lupus erythematosus and risk of sexual dysfunction: a systematic review and meta-analysis. Lupus. 2021;30(2):238–47. 10.1177/0961203320974081 33210559

[5] Bode C, van der Heij A, Taal E, et al. Body-self unity and self-esteem in patients with rheumatic diseases. Psychol Health Med. 2010;15(6):672–84. 10.1080/13548506.2010.507774 21154020

[6] Carrillo A, Marengo-Rodríguez D, Ibarra-Velasco-Siles M, et al. Addressing the unspoken: sexual dysfunction in men with systemic lupus erythematosus, a call to action for rheumatologists. Sex Med Rev. 2024;12(3):434–41. 10.1093/sxmrev/qeae033 38796305

[7] Lotti F, Maggi M. Sexual dysfunction and male infertility. Nat Rev Urol. 2018;15(5):287–307. 10.1038/nrurol.2018.20 29532805

[8] Raina R, Pahlajani G, Khan S, et al. Female sexual dysfunction: classification, pathophysiology, and management. Fertil Steril. 2007;88(5):1273–84. 10.1016/j.fertnstert.2007.09.012 17991514

[9] Serna-Peña G, Colunga-Pedraza IJ, Villarreal-Alarcón MÁ, et al. Sexual function in women with systemic lupus erythematosus: a case-control study. Rheumatol Int. 2021;41(8):1465–9. 10.1007/s00296-021-04895-3 34021365

[10] García Morales M, Callejas Rubio JI, Peralta-Ramírez MI, et al. Impaired sexual function in women with systemic lupus erythematosus: a cross-sectional study. Lupus. 2013;22(10):987–95. 10.1177/0961203313500370 23963430

[11] Zhang L, Fu T, Yin R, et al. Prevalence of depression and anxiety in systemic lupus erythematosus: a systematic review and meta-analysis. BMC Psychiatry. 2017;17(1):70. 10.1186/s12888-017-1234-1 PMC5310017 28196529

[12] Cornwell CJ, Schmitt MH. Perceived health status, self-esteem, and body image in women with rheumatoid arthritis or systemic lupus erythematosus. Res Nurs Health. 1990;13(2):99–107. 10.1002/nur.4770130206 2320762

[13] Minopoulou I, Pyrgidis N, Tishukov M, et al. Sexual dysfunction in women with systemic autoimmune rheumatic disorders: a systematic review and meta-analysis. Rheumatology (Oxford). 2023;62(3):1021–30. 10.1093/rheumatology/keac457 35951753

[14] Perez-Garcia LF, Te Winkel B, Carrizales JP, et al. Sexual function and reproduction can be impaired in men with rheumatic diseases: a systematic review. Semin Arthritis Rheum. 2020;50(3):557–73. 10.1016/j.semarthrit.2020.02.002 32165034

[15] Aringer M, Costenbader K, Daikh D, et al. 2019 European League Against Rheumatism/American College of Rheumatology classification criteria for systemic lupus erythematosus. Arthritis Rheumatol. 2019;71(9):1400–12. 10.1002/art.40930 PMC6827566 31385462

[16] Shen B, He Y, Chen H, et al. Body image disturbances impact sexual problems in Chinese systemic lupus erythematosus patients. J Immunol Res. 2015;2015:204513. 10.1155/2015/204513 PMC4451262 26090484

[17] Peris M, Maganto C, Garaigordobil M. Escala de autoestima corporal: datos psicométricos de fiabilidad y validez. Revista de Psicología Clínica con Niños. y Adolescentes. 2016;3(2):51–8.

[18] Rosenberg M. Society and the adolescent self-image. Princeton, NJ: Princeton University Press; 1965.

[19] Martín-Albo J, Núñez JL, Navarro JG, et al. The Rosenberg Self-Esteem Scale: translation and validation in university students. Span J Psychol. 2007;10(2):458–67. 10.1017/s1138741600006727 17992972

[20] Rosenberg M. Conceiving the self. New York: Basic Books; 1979.

[21] Garcia-Portilla MP, Saiz PA, Fonseca E, et al. Psychometric properties of the Spanish version of the Changes in Sexual Functioning Questionnaire Short-Form (CSFQ-14) in patients with severe mental disorders. J Sex Med. 2011;8(5):1371–82. 10.1111/j.1743-6109.2010.02043.x 20946156

[22] Clayton AH, McGarvey EL, Clavet GJ. The Changes in Sexual Functioning Questionnaire (CSFQ): development, reliability, and validity. Psychopharmacol Bull. 1997;33(4):731–45. 9493486

[23] Gladman DD, Ibanez D, Urowitz MB. Systemic lupus erythematosus disease activity index 2000. J Rheumatol. 2002;29(2):288–91. 11838846

[24] Gladman DD, Goldsmith CH, Urowitz MB, et al. The systemic lupus international collaborating clinics/American College of Rheumatology (SLICC/ACR) damage index for systemic lupus erythematosus: international comparison. J Rheumatol. 2000;27(2):373–6. 10685799

[25] Xia XY, Liu ST, Song ZC, et al. Current investigation of the high prevalence of sexual dysfunction in female patients with systemic lupus erythematosus: a cross-sectional study. Rheumatol Int. 2024;44(10):2147–57. 10.1007/s00296-024-05690-6 39153077

[26] Gebeyehu NA, Gesese MM, Tegegne KD, et al. Global prevalence of sexual dysfunction among diabetic patients from 2008 to 2022: systematic review and meta-analysis. Metabol Open. 2023;18:100247. 10.1016/j.metop.2023.100247 PMC10267599 37323562

[27] Rutte A, van Splunter MMI, van der Heijden AAWA, et al. Prevalence and Correlates of Sexual Dysfunction in Men and Women With Type 2 Diabetes. J Sex Marital Ther. 2015;41(6):680–90. 10.1080/0092623X.2014.966399 25256659

[28] Mann M, Hosman CMH, Schaalma HP, et al. Self-esteem in a broad-spectrum approach for mental health promotion. Health Educ Res. 2004;19(4):357–72. 10.1093/her/cyg041 15199011

[29] Katz MR, Rodin G, Devins GM. Self-esteem and cancer: theory and research. Can J Psychiatry. 1995;40(10):608–15. 10.1177/070674379504001007 8681258

[30] Chen H, Shi S, Cui H, et al. Predictive factors of body image disturbance in patients with systemic lupus erythematosus: a cross-sectional study in China. BMJ Open. 2022;12(11):e060049. 10.1136/bmjopen-2021-060049 PMC9703306 36428020

[31] Jolly M, Simon Pickard A, Mikolaitis RA, et al. Body image in patients with systemic lupus erythematosus. Int J Behav Med. 2012;19(2):157–64. 10.1007/s12529-011-9154-9 21380770

[32] Jones MM, Kimble LP. Body Image, Depression Symptoms, and Health-Related Quality of Life in Black Women With Systemic Lupus Erythematosus. Nurs Womens Health. 2022;26(5):363–70. 10.1016/j.nwh.2022.07.008 PMC9659411 36002068

[33] Sutanto B, Singh-Grewal D, McNeil HP, et al. Experiences and perspectives of adults living with systemic lupus erythematosus: thematic synthesis of qualitative studies. Arthritis Care Res (Hoboken). 2013;65(11):1752–65. 10.1002/acr.22032 23609952

[34] Woertman L, van den Brink F. Body image and female sexual functioning and behavior: a review. J Sex Res. 2012;49(2–3):184–211. 10.1080/00224499.2012.658586 22380588

[35] Satinsky S, Reece M, Dennis B, Sanders S, Bardzell S. An assessment of body appreciation and its relationship to sexual function in women. Body Image. 2012;9(1):137–44. 10.1016/j.bodyim.2011.09.007 22018776

[36] Tafarodi RW, Swann WB Jr. Self-liking and self-competence as dimensions of global self-esteem: initial validation of a measure. J Pers Assess. 1995;65(2):322–42. 10.1207/s15327752jpa6502_8 8656329

[37] Schmitt DP, Allik J. Simultaneous administration of the Rosenberg Self-Esteem Scale in 53 nations: exploring the universal and culture-specific features of global self-esteem. J Pers Soc Psychol. 2005;89(4):623–42. 10.1037/0022-3514.89.4.623 16287423

[38] Leon L, Clemente D, Heredia C, et al. Self-esteem, self-concept, and body image of young people with rheumatic and musculoskeletal diseases: A systematic literature review. Semin Arthritis Rheum. 2024;68:152486. 10.1016/j.semarthrit.2024.152486 38896913

[39] Daleboudt GM, Broadbent E, McQueen F, et al. The impact of illness perceptions on sexual functioning in patients with systemic lupus erythematosus. J Psychosom Res. 2013;74(3):260–4. 10.1016/j.jpsychores.2012.11.004 23438719

[40] Nieto R, Quintana R, Zavala-Flores E, et al. Time to diagnosis in systemic lupus erythematosus: Associated factors and its impact on damage accrual and mortality. Data from a multi-ethnic, multinational Latin American lupus cohort. Lupus. 2024;33(4):340–6. 10.1177/09612033241232821 38334100

[41] Ugarte-Gil MF, Alarcón GS. Systemic lupus erythematosus in Latin America: Outcomes and therapeutic challenges. Clin Immunol Commun. 2023;4:60–4.

[42] Liu M, Dou J, Wang Q. The effect of systemic lupus erythematosus on sexual function in women: an updated meta-analysis based on cross-sectional studies. Adv Rheumatol. 2022;62(1):24. 10.1186/s42358-022-00257-0 35787296

[43] Brouwer J, Hazes JM, Laven JS, et al. Fertility in women with rheumatoid arthritis: influence of disease activity and medication. Ann Rheum Dis. 2015;74(10):1836–41. 10.1136/annrheumdis-2014-205383 24833784

[44] Campos-Guzmán J, Valdez-López M, Govea-Peláez S, et al. Determinants of sexual function in male patients with systemic lupus erythematosus. Lupus. 2022;31(10):1211–7. 10.1177/09612033221107802 35702930

[45] Merayo-Chalico J, Barrera-Vargas A, Morales-Padilla S, et al. Epidemiologic Profile of Erectile Dysfunction in Patients with Systemic Lupus Erythematosus: The Latin American Landscape. J Rheumatol. 2019;46(4):397–404. 10.3899/jrheum.180292 30647184

[46] Sinclair SJ, Blais MA, Gansler DA, et al. Psychometric properties of the Rosenberg Self-Esteem Scale: overall and across demographic groups living within the United States. Eval Health Prof. 2010;33(1):56–80. 10.1177/0163278709356187 20164106

[47] Burke SM, Adnan Majid DS, Manzouri AH, et al. Sex differences in own and other body perception. Hum Brain Mapp. 2019;40(2):474–88. 10.1002/hbm.24388 PMC6587810 30430680

[48] Hidalgo DA, Dewitte M. Individual, Relational, and Sociocultural Determinants of Sexual Function and Sexual Satisfaction in Ecuador. Sex Med. 2021;9(2):100307. 10.1016/j.esxm.2020.100307 PMC8072141 33721691

[49] Rodrigues L, Sim-Sim MMF, Sousa L, et al. Self-concept and body image of people living with lupus: A systematic review. Int J Rheum Dis. 2021;24(11):1339–53. 10.1111/1756-185X.14187 34314107

[50] Morales-Sánchez L, Luque-Ribelles V, Gil-Olarte P, et al. Enhancing Self-Esteem and Body Image of Breast Cancer Women through Interventions: A Systematic Review. Int J Environ Res Public Health. 2021;18(4):1640. 10.3390/ijerph18041640 PMC7914971 33572137

[51] Velasco L, Gutiérrez L, Alcocer N, et al. Exploring the moderated role of two psychological programs on sexual pleasure from physical symptoms, body image distortion, and emotional problems of Hispanic women with breast cancer. J Sex Marital Ther. 2023;49(6):643–58. 10.1080/0092623X.2023.2173111 36735842

[52] Martínez-Jabaloyas JM, Moncada I, Rodríguez-Vela L, et al. Evaluation of self-esteem in males with erectile dysfunction treated with Viagra. Analysis of a Spanish patients group selected from a multicenter, international study. Actas Urol Esp. 2010;34(8):699–707. 20800034

